# A model of Periventricular Leukomalacia (PVL) in neonate mice with histopathological and neurodevelopmental outcomes mimicking human PVL in neonates

**DOI:** 10.1371/journal.pone.0175438

**Published:** 2017-04-13

**Authors:** Nahla Zaghloul, Hardik Patel, Mohamed Nagy Ahmed

**Affiliations:** 1 Department of Pediatrics, Division of Neonatology, Cohen Children’s Medical Center of New York, New Hyde Park, New York, United States of America; 2 Feinstein institute for Medical Research, Manhasset, New York, United States of America; Instituto Cajal-CSIC, SPAIN

## Abstract

Periventricular leukomalacia (PVL), a brain injury affecting premature infants is commonly associated with cerebral palsy. PVL results from hypoxia-ischemia (HI) with or without infection and is characterized by white matter necrotic lesions, hypomyelination, microglial activation, astrogliosis, and neuronal death. It is important to study a PVL mouse model that mimics human PVL in symptomatology, anatomic and molecular basis. In our neonate mice model, bilateral carotid arteries were temporary ligated at P5 followed by hypoxic exposure (FiO_2_ of 8% for 20 min.). At P5 in mice, the white matter is more vulnerable to HI injury than the grey matter. In our PVL model, mice suffer from significant hind limb paresis, incoordination and feeding difficulties. Histologically they present with ventriculomegally, white matter loss, microglial activation and neuronal apoptosis. HI injury increases proinflammtory cytokines, activates NF-kB, activates microglia and causes nitrative stress. All these inflammatory mediators lead to oligodendroglial injury and white matter loss. Neurobehavioral analysis in the PVL mice model at P60 showed that the HI group had a significant decrease in hind limb strength, worsening rotarod testing and worsening performance in the open field test. This new PVL model has great advantages far beyond just mimicking human PVL in clinical features and histopathology. Long term survival, the development of cerebral palsy and the ability of using this model in transgenic animals will increase our understanding of the mechanistic pathways underlying PVL and defining specific targets for the development of suitable therapeutics.

## Introduction

Periventricular leukomalacia (PVL) is a major neuropathological white matter brain injury that is closely associated with cerebral palsy (CP) in premature infants. Risk factors for the development of PVL include: prematurity associated with immature cerebrovascular development, hypoxic-ischemic (HI) insults with lack of appropriate auto-regulation of cerebral blood flow, free radical production, energy deprivation, intrauterine infection and chorioamnionitis. Affected infants show definitive signs of cerebral palsy such as spastic diplegia and other signs of cerebral injury associated with PVL, including: seizures, developmental delay, visual and hearing impairment, scoliosis or incontinence by 6–9 months of age [1]. In US, about 60,000 very low birth weight infants (<1500 g) are born annually [2]. While almost 90% of these infants survive, approximately 10% of the survivors show signs of CP and 25–50% display cognitive or behavioral deficits [2]. In extremely low birth weight infants (birth weight<1000 g), the incidence of CP is estimated to be in 20% of survivors [2].

PVL pathology is characterized by focal necrosis, marked astrogliosis, microgliosis and a decrease in pre-myelinating oligodendrocyte precursor cells. The loss of these oligodendrocyte precursors leads to a compensatory generation of additional or newly replicated oligodendrocyte precursors that have a limited capacity for differentiation, that ultimately leads to hypomyelination and thus ventriculomegaly. Candidate pathological mechanisms that drive white matter injury leading to oligodendroglial loss and death include oxidative stress, excitotoxicity, and inflammation [3–5]. In neonatal rat models of PVL, microglia exhibit a robust inflammatory response associated with white matter injury [1]. Microglial density increases rapidly within 24 h after HI and continues to rise until 96 h post HI [1].

PVL animal models are induced by HI, infection/inflammation and excitotoxicity. HI models were first introduced in an immature rat model and consisted of unilateral carotid ligation and exposure to 8% oxygen for 1–3 hours [6]. Most studies which use this model, have focused on gray matter injury. Using this model in 1 day old rats produced predominantly grey matter injury [7,8]. This model has also been used in 7 and 9 day old mice to study the response of immature oligodendroglial and stem/progenitor cells in white matter and periventricular zones.[8–11]. A recent modification of this model was used in 7 day old rats which underwent unilateral carotid artery ligation followed by hypoxic exposure (FiO2 of 6%, for 1 hour). In this model, there was a selective loss of O1+ oligodendroglia and a decrease of MBP in the corpus callosum and periventricular white matter 4 days later, without affecting gray matter [8,12]. A rat model of permanent bilateral carotid artery ligation and 10-min exposure to 8% oxygen at PND 7 showed a loss of O4+ cells in the corpus callosum [8,13]. Permanent bilateral carotid artery ligation of PND5 or PND7 rats resulted in subcortical white and gray matter injuries as shown in many studies but present with limited survival beyond 2–3 days which limit assessment of long term outcome [8,11,14]. In contrast, permanent bilateral carotid artery ligation of 1-day-old rats allowed long term survival longer than 2 weeks and produced white matter injury (WMI) in corpus callosum, subcortex, internal capsule, and a significant enlargement of the ventricles with limited pathology in the gray matter [15]. A two-hour transient bilateral carotid ligation produced similar distribution of damage in the subcortical regions and allowed survival of more than 2 weeks [15]. Interestingly, a model was developed in rabbit fetuses of gestational days 21–25 subjected to intrauterine hypoperfusion, showed WMI 1–7 days after the insult [8,16]. Post-natal changes were found in the white matter on MRI and neurological dysfunctions were demonstrated 10 days post intrauterine hypoperfusion [8,16].

In this study we aimed to establish a new PVL model, in neonate mice, which had the histopathological (white matter injury) and clinical features of human PVL. This new model had to mimic human PVL, not only in imaging and pathology, but in long term neurodevelopmental outcome where a subset of these animals developed cerebral palsy. We also wanted the model to survive many month post HI. The advantages of using a mouse model of PVL are cost efficiency, availability of antibodies and transgenic animals which can be used in both mechanistic and therapeutic studies. Long term survival of this model up to 6 month after HI will allow better understanding of different mechanisms involved in PVL pathogenesis and the testing of various prophylactic and therapeutic interventions and outcome monitoring.

## Material and methods

### Animals

All procedures were performed in accordance with the NIH Guidelines on the care and use of vertebrate animals and approved by the Institutional Animal Care and Use Committee of the Feinstein Institute for Medical Research. Wild type C57BL/6 pups were used.

### Hypoxia-ischemia insult

Under aseptic conditions, P5 pups were anaesthetized with isoflurane 2%. A vertical midline neck incision was performed and both carotid arteries were temporarily ligated for10 minutes using 6.0 silk sutures. Following ischemic insult the sutures were removed and neck incision closed. Pups were allowed to recover for 30 min. on thermal blanket. After recovery, they were placed in hypoxia chamber (8% O_2_) for 20 min. after which they were returned to their dams. Sham controls at P5 received isoflurane anesthesia, a vertical midline neck incision followed by repair. Both carotid arteries were isolated but not ligated [13]. Pups were monitored during the procedure and for 4 hours thereafter, then twice daily for the first week then daily thereafter. 6% of animals became severely ill during the procedure and within 3 days thereafter. Moribund animals showing labored breathing, lethargy, severe bleeding or severe dehydration underwent euthanasia.

### Histopathology and immunohistochemistry

Animals were deeply anesthetized with a lethal dose of xylazene/ketamine and perfused transcardially with saline, then 4% paraformaldehyde (PFA). Whole brain tissue was fixed in 4% PFA for 24h, processed, paraffin embedded and sectioned coronally at 6 μm thick. Following deparaffinization, hematoxylin and eosin (H&E) staining for the cortex and hippocampus were performed according to standard protocols. N = 12 animals/group. For immunofloresence, following deparaffinization and antigen retrieval, sections were incubated for 2h at room temperature in TBS+ 1% Triton-X + 10% donkey serum. Sections were incubated for 24h at 4°C with primary antibodies, followed by 2h incubation at RT with the appropriate secondary antibody with DAPI. All images were captured on a confocal microscope (Olympus Fluoview 300 Confocal Microscope). The following primary antibodies were used to detect the following markers: Arginase 1 (Santa Cruz Biotechnology {1:50}, Dallas, TX, USA); CD 68 (AbS Serotec {1:100}, Raleigh, NC); Cleaved Caspase 3 (Cell Signaling Technology {1:50},Danvers, MA, USA); GFAP (Abcam {1:500} Cambridge,MA, USA); Iba1 (Wako {1:400}, Richmond, VA, USA); CNPase (Abcam {1:200} Cambridge,MA, USA); NeuN (EDM Millipore {1:250} Billerica, MA, USA); Nitrotyrosine (Abcam {1:200} Cambridge,MA, USA); Olig2 (Santa Cruz Biotechnology{1:50} Dallas, TX, USA) and secondary antibodies (Species specific Cy3 and FITC 1:125 Jackson Immunoresearch, Westgroove, PA).

#### Immunostaining analysis

Digital images were obtained using Confocal software and were exported to Image J. The fluorescence intensity associated with each pixel was determined in 5 animals per group with 4 sections per animal. Each section corresponds to 750 x750 μm. Excitation and acquisition parameters were adjusted to fully eliminate pixel saturation and all images were collected under identical settings. Cell counting was performed on 4 sections per animal (750 x750 μm each) and 5 animals per group.

### Inflammatory cytokines assay

Assay of inflammatory cytokines; IL1, IL6 and CXCL2 were performed using Quantikine ELISA kits (R&DSystems, Minneopolis, MN) per manufacturer instructions. N = 8 animals/group.

### Western blot

After protein extraction, protein concentration was estimated using the Modified Lowry Protein Assay (Thermo Fisher Scientific, Rockford, IL, USA). Standard SDS-PAGE techniques were followed. After electrophoresis, proteins were transferred to a PVDF membrane using a Wet/Tank Blotting System (Bio-rad, Hercules, CA, USA). Membranes were briefly washed, incubated with respective primary antibody in 5% BSA with PBST overnight. After washing, the membranes were incubated with HRP-conjugated secondary antibodies for 60 min, washed, processed using Amersham ECL detection systems (GE healthcare, Piscataway, NJ USA) and exposed to 8×10 Fuji X-Ray Film. The density of each band was presented as a ratio in comparison to Actin band density. The following primary antibodies were used: Cleaved Caspase 3 (Cell Signaling Technology {1:500}, Danvers, MA, USA); phospho p65 (Cell Signaling Technology {1:500}, Danvers, MA, USA); P65 (Cell Signaling Technology {1:500}, Danvers, MA, USA); iNOS (Cell Signaling Technology {1:500}, Danver, MA); eNOS (Cell Signaling Technology {1:500}, Danver, MA, USA); nNOS (Cell Signaling Technology {1:500}, Danver, MA); and anti- Beta -Actin protein (as an internal control) (Cell Signaling Technology {1:1000}, Danvers, MA, USA).

Horseradish Peroxidase (HRP)-Conjugated Goat Anti-Rabbit IgG conjugate was used for detection of rabbit primary antibodies (Bio-Rad {1:5,000}, Hercules, CA, USA). Goat anti-mouse HRP conjugates were used for detection of mouse primary antibodies (Southern Biotech {1:5,000}, Birmingham, AL, USA). N = 6 animals/group.

#### MRI instrumentation and data acquisition and analysis

MRI data were obtained using 9.4/30 BioSpect Spectrometer (Bruker BioSpin Corp., Germany) equipped with 72 mm volume coil as a transmitter and 4-channel mouse brain coil. High resolution RARE T2-weighted images in axial and coronal plain were acquired using the following parameters: TR/TE = 3524/36 ms, FOV = 1.5 cm, matrix = 256x256, 0.5 mm slice thickness, Nslices = 30, Navg = 4, and RARE Factor = 8.

Anesthetized animals (1–2% isoflurane with carbogen (95% O_2_+5% CO_2_) were placed in the MRI unit. Their respiration and temperature were continuously monitored using Small Animal Instruments monitoring system (Small Animals Instruments, Inc. stony Brook, NY).

#### Image post processing

The axially acquired T2-weighted images (0.5 mm slice thickness) were used for data analysis. Mask of the mouse brain and ventricle were obtained by manually tracing area of interest. Coronally acquired images were used for conformation purposes. Images were then imported into Analyze 7.5 software (Biomedical Imaging Resource, Mayo Clinic, Rochester, MN) for manual and semi-automated volume rendering. We estimated brain volumes for the 0.5 mm gap using Analyze. Total brain tissue volume was defined as the total intracranial volume minus all cerebrospinal fluid (CSF) spaces. N = 10 animals/group.

### Neurobehavioral testing and long term outcome

Testing of motor function was performed by using a rotarod device (Columbus Instruments, Columbus, OH) at 60 days of age. Each session consisted of three trials that were averaged on the elevated accelerating rotarod beginning at 5 r.p.m./minute, in a trial to measure the time through which, the mouse was able to remain on the rod. Grip strength measurements for forelimb and hindlimb were tested using a grip strength meter (Columbus Instruments). Each session consisted of three tests per animal and the values were averaged. At P60, animals were tested in an open field analysis (San Diego Instruments, San Diego, CA). Animals were given several minutes to adapt to the testing chamber before the beginning of testing. Open field data was digitally recorded for 30 minutes and subsequently analyzed by Noldus Ethovision tracking software [17]. Beam breaks were recorded in the x, y, and z planes and averaged across groups. N = 25 animals/group.

### Statistical analysis

All statistical tests were performed with Graph Pad Prism 6 software (La Jolla, CA). Statistical analysis of mean differences between groups was performed by Student’s t test. All P values and N values are indicated in figure legends.

## Results

### PVL model

**Fig 1:**
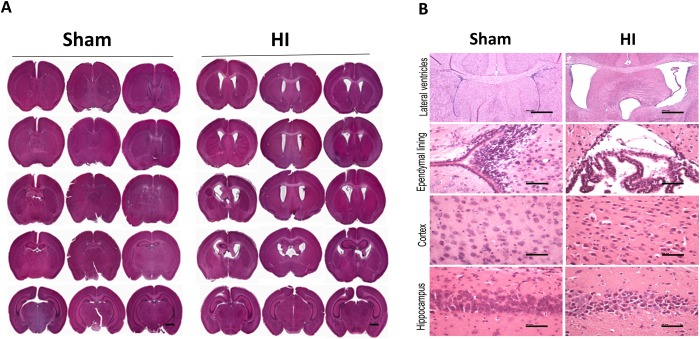
Histopathological studies. **Panel A:** Serial H&E coronal whole brain sections from anterior (top) to posterior (bottom) of 3 randomly selected animals from each group Sham and HI group, with special emphasis on lateral and 3^rd^ ventricle size. Scale bar 2 mm. N = 12 animals/group. **Panel B:** Representative H&E coronal brain sections of lateral ventricle, ependymal lining, cortex and hippocampus of the studied groups (Sham & HI group). Scale bar 10 μm. N = 12 animals/group.

PVL neonate mice displayed characteristic ventricular enlargement likely resulting from cortical white matter loss shown by histopathological studies of hemotoxylin and eosin stained serial coronal sections spanning the lateral ventricles (Fig 1). The PVL mouse model showed marked enlargement of both lateral ventricles and third ventricle (Fig 1A). The ependymal lining was thickened and showed increased evidence of injury in the form of mitosis (Fig 1B). There was neuronal damage in cortex and hippocampus in the HI injured group (Fig 1B).

### MRI studies

**Fig 2:**
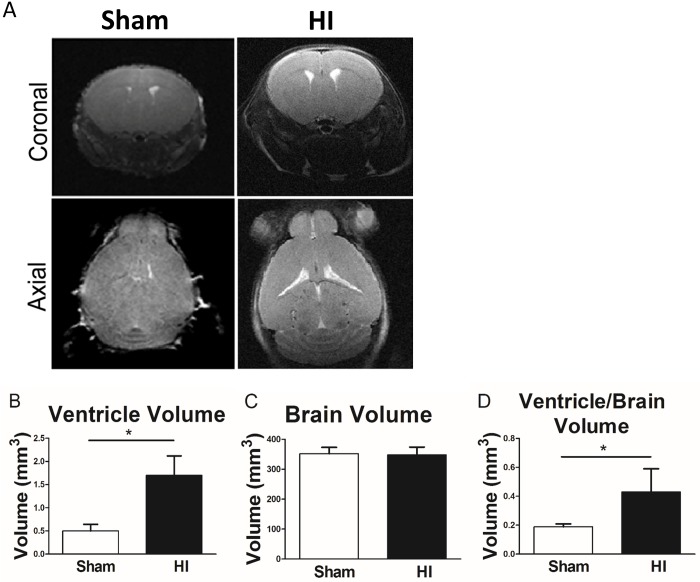
MRI volumetric analysis in both studied groups (Sham and HI group), using the BioSpec 94/30 imaging system is a 9.4T horizontal bore magnet operates at 400 MHz and runs ParaVision^™^ 4.0 software. **Panel A:** Coronal (top) and axial (bottom) brain slices. **Panel B:** lateral ventricle volume bilaterally measured by paravision software in both studied groups. **Panel C:** Brain volume includes brain volume + lateral ventricle volume measured by paravision software in the studied groups. **Panel D:** The ratio of bilateral lateral ventricle volume to total brain volume measured by paravision software in the studied groups. Since there was no significant difference in brain volume, increased lateral ventricle volume indicates white matter loss. N = 10 animals/group. Bars represent the Mean + SE. * indicates P< 0.05.

Brain MRI imaging at P15 (10 days post HI) using the BioSpec 94/30 Imaging 9.4T showed enlarged lateral ventricles in HI injured group (Fig 2A). Summary data from MRI imaging demonstrate a significant increase of lateral ventricle volume in HI injured as compared to sham (P<0.05) (Fig 2B). There was no significant difference in total brain volume between the two groups (Fig 2C). The ratio of ventricle volume to total brain volume was significantly increased in HI group as compared to sham (P<0.05) (Fig 2D).

### Myelination process

**Fig 3:**
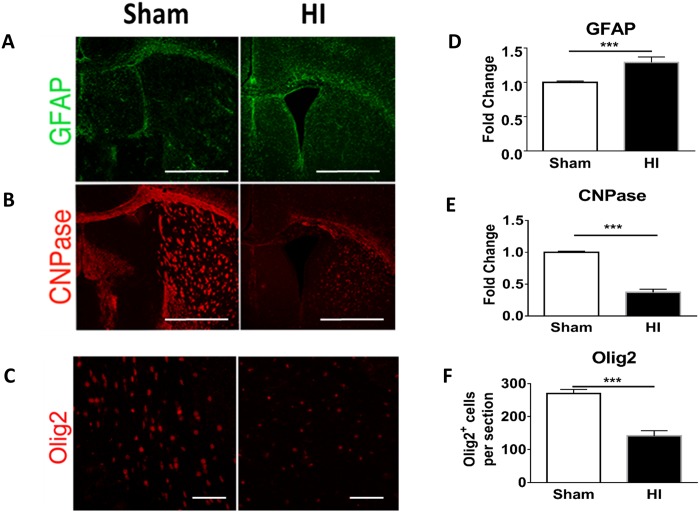
Astrogliosis and white matter volume loss in PVL model. Immunohistochemistry of periventricular brain area at P15 (or 10 days after HI) in both studied groups (Sham vs HI group). **Panel A:** Astrocytes (GFAP) in green, **Panel B:** Oligodendroglia (CNPase) in red. **Panel C**: Olig 2 (oligodendroglia) in red. Scale bar = 100 μm. **Panel D**: Quantification of GFAP intensity as fold change where sham = 1. **Panel E**: Quantification of CNPase intensity as fold change where sham = 1. **Panel F**: Quantification of Olig2 positive cells per section. There was obvious significant reduction of oligodendroglial cells (indicating white matter loss), accompanied with significant increase of astrocytes in HI group. N = 5 animals/group. 4 sections /animal. Bars represent the Mean + SE. ** * indicates P< 0.001.

To determine the effect of HI on myelination, we assessed levels of 2',3'-Cyclic-nucleotide 3'-phosphodiesterase (CNPase) and Olig 2. Both CNPase and Olig2 levels were significantly decreased in HI group as compared to sham (Fig 3B, 3C, 3E and 3F).

### Astrocytes

There was significant increase in astrocytes in the periventricular area of HI group as compared to sham (Fig 2A and 2D).

### Apoptosis

**Fig 4:**
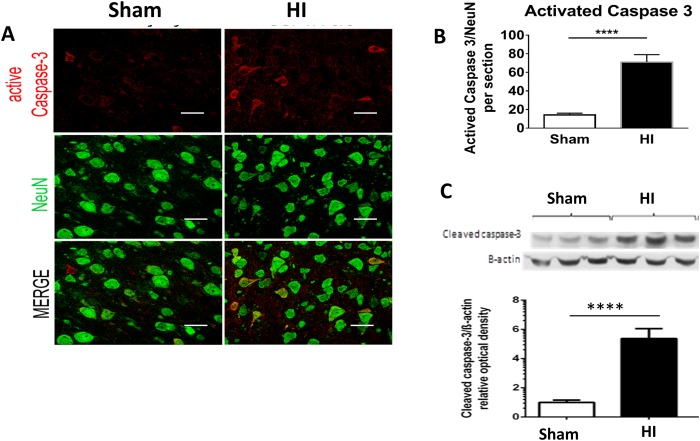
Neuronal apoptosis in PVL model. **Panel A:** Immunohistochemistry of periventricular brain area at P15 (or 10 days after HI) of the 2 studied groups (Sham and HI group). **Panel A**: Apoptosis (Caspase 3) shown in red (top), Neurons (NeuN) shown in green (middle). Co-localization is shown in orange indicates neuronal apoptosis (bottom). Scale bar = 50 μm. **Panel B:** Activated Caspase 3/ NeuN quantification. N = 5 animals/group & 4 sections/animal. Bars represent the Mean + SE. ** * *indicates P< 0.0001. **Panel C:** Western blot of periventricular area of brain protein lysate at P10 (or 10 days after HI) in both studied groups. The western blot was probed for Cleaved caspase 3 (top) and reprobed for β Actin (bottom) as loading controls. Fold change of the western blot determined using Image J to measure band intensities of cleaved caspase 3 normalized to β-Actin. Caspase 3 is upregulated by 5.4 fold in HI group as compared to sham. N = 6 animals/group. Bars represent the Mean + SE. ** * *indicates P< 0.0001.

For further evaluation of the neuronal damage seen on H&E in the cortex and hippocampus, we stained for apoptosis marker. Quantitative and qualitative assessment of Caspase 3, showed a significant increase in HI model and was co-localized with neurons indicating neuronal apoptosis (Fig 4A and 4B). Western blot for cleaved caspase 3 of the periventricular area of brain at P10 showed significant increase in HI group as compared to sham (Fig 4C)

### Microglial activation and inflammatory markers

**Fig 5:**
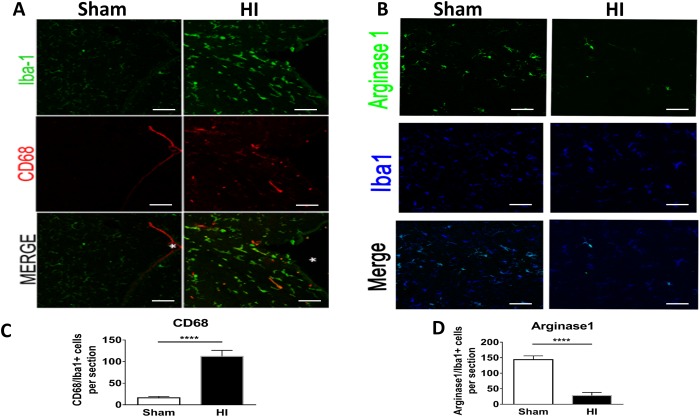
Studies of microglial M1 and M2 phenotypes at P15 (10 days after HI) in HI group versus sham. **Panel A:** Immunostaining for all Microglia (Iba1) in green (top), CD 68 (marker of activated microglia only) in red (middle). Co-localization in yellow indicating amount of activated microglia. Scale Bar = 100 μm. **Panel B:** Immunostaining for Arginase 1 (M2 microglial marker) in green (top); for Microglia (Iba1) in blue (middle); and Co-localization (bottom) indicating the percentage of M2 microglia. Co-localization showed scare M2 microglial phenotype cells in HI group. Scale Bar = 100 μm. **Panel C**: Quantification of CD68/Iba1 per section **Panel D**: Arginase 1/Iba1 per section. There was significant increase of M1 microglia and a significant reduction of M2 microlgia in HI group. N = 5 animals/group & 4 sections/animal. Bars represent the Mean + SE. **** indicates P< 0.0001.

We hypothesized that the white matter injury in HI group was due to accentuation of the pro-inflammatory microglial response upon activation. Therefore, we evaluated CD68 expression, a marker for activated pro-inflammatory microglia. CD68+ microglia near lateral ventricles were significantly increased as compared to sham (Fig 5A and 5C). Arginase 1, a marker of repair microglia was significantly reduced in the HI model as compared to sham (Fig 5B and 5C).

### Nitrative stress

**Fig 6:**
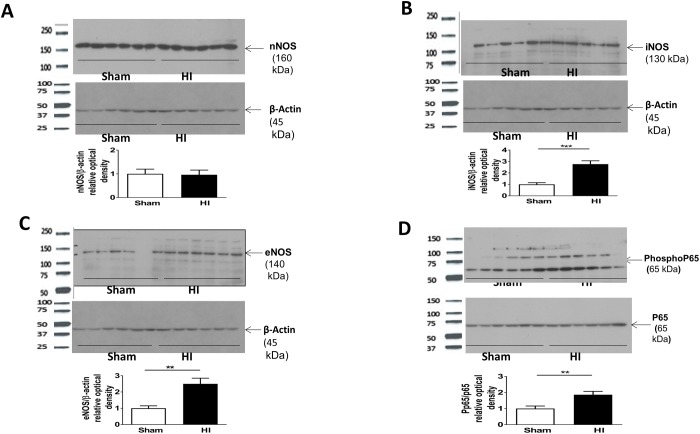
Nitric oxide synthetase isoforms and NF-kb activation in PVL model. **Panel A:** nNOS western blot (MW 160 kDa) of periventricular area of brain protein lysate at P10 (or 5 days after HI) in both studied groups and represented as a ratio for β-Actin protein. No difference. **Panel B:** iNOS western blot (MW 130 kDa) of periventricular area of brain protein lysate at P10 (or 5 days after HI) in both studied groups and represented as a ratio for β –Actin protein. **Panel C:** eNOS western blot (MW 140 kDa) of periventricular area of brain protein lysate at P10 (or 5 days after HI) in both studied groups and represented as a ratio for B-Actin protein. **Panel D:** Phosphorylated P65 western blot (MW 65 kDa) of periventricular area of brain protein lysate at P10 (or 5 days after HI) in both studied groups and represented as a ratio to p65 protein (MW 65 kDa). **For Panels A, B, C, D:** N = 6 animals/group. Bars represent the Mean + SE. * indicates P< 0.05. ** indicates P< 0.01. *** indicates P< 0.001.

**Fig 7:**
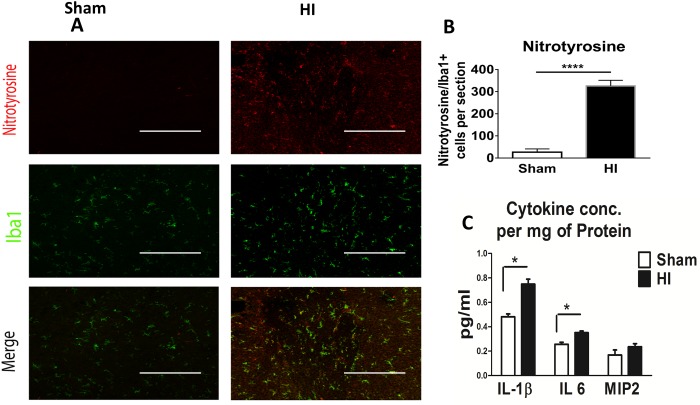
Nitrative stress and pro-inflammatory cytokines. **Panel A:** Immunohistochemistry of coronal sections of the periventricular brain area of postnatal day 15 pups (or 10 days after HI) of the two studied groups (sham and HI group). Top panel shows Nitrotyrosine (marker of nitrative stress) in red. Middle panel shows Iba1 (microglial marker) in green. Lower panel shows Co-localization in yellow nitrotyrosine secreted by microglia. There was a significant increase of nitrative stress in HI group compared to sham. Scale bar = 200 μm. **Panel B:** Quantification of Nitrotyrosine/Iba1 per section. N = 5 animals/group & 4 sections/animal. Bars represent the Mean + SE. **** indicates P< 0.0001. **Panel C:** ELISA assay of cytokines known to peak in both human and animal PVL model. Assay from brain homogenate at P6 or 24 hrs post HI, namely IL-1β, IL 6, MIP2, of the studied groups (Sham and HI group). There was a significant increase of both IL1β & IL6 in HI group compared to sham. N = 8 animals/group. Bars represent the Mean + SE. * indicates P< 0.05.

A mechanism of injury to the developing oligodendrocytes (OLs) that underlies the pathogenesis of PVL and the hypomyelination seen in PVL is mediated by exposure to reactive oxygen and nitrate species [4,18]. Nitrative stress was confirmed by western blot of the periventricular area of brain at P10 which showed a significant increase of both inducible and endothelial nitric oxide synthetase protein expression (P<0.05) (Fig 6B and 6C) but not the neuronal isoform of nitric oxide synthetase in HI model as compared to sham (Fig 6A). In human PVL, free radical injury leads to increased lipid peroxidation and nitrotyrosine production. Nitrotyrosine is produced by microglia as shown by the co-localization of Nitrotyrosine with Iba 1 (Fig 7A). Nitrotyrosine staining is significantly increased in HI as compared to sham (Fig 7B).

### NF- kB activation and pro-inflammatory cytokines

Phosphorylated p65/p65 was significantly increased in the periventricular area of brain at P10 in HI group as compared to sham (P<0.05) indicating activation of NF-kB pathway (Fig 6D).

Additionally, pro-inflammatory mediators (IL1β, IL6) were significantly increased in brain homogenate 24 hours following HI injury (Fig 7C).

### Long term outcome

**Fig 8:**
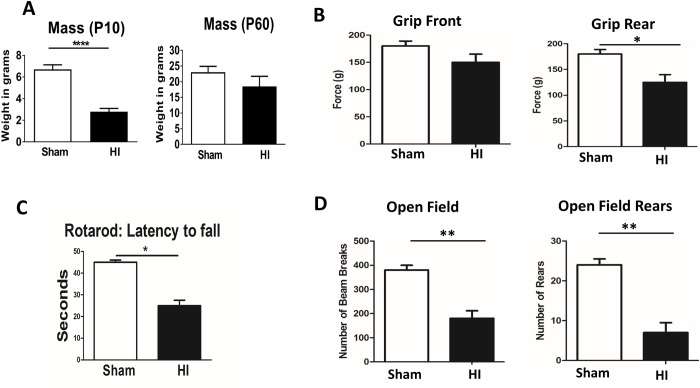
Long term outcome assessed by neurobehavioral testing performed at postnatal day 60. **Panel A:** There was a reduction of total body mass at P10 (P<0.0003), but the difference was not significant at P60. **Panel B:** Testing for grip, showed no difference in front grip between the 2 groups, while rear grip strength was significantly lower in HI group compared to sham (P<0.05). **Panel C:** A significant reduction of latency to fall indicating worse co-ordination was observed in HI group compared to sham (P<0.05). **Panel D:** Number of beam breaks and number of rears in the open field test were significantly reduced in HI group as compared to sham (P<0.05). N = 25 animals/group. Bars represent the Mean + SE. ** indicates P< 0.01.

At P60 neurobehavioral analysis was performed. At P60 there was no significant difference in body weights between the 2 groups. Initially body weight was significantly lower in HI group due to impaired feeding (Fig 8A). Rear grip strength was significantly decreased in HI group (Fig 8B). On rotarod, HI group had a more tendency and shorter latency to fall indicating impaired co-ordination (Fig 8C). By analyzing open field, number of rears and beam breaks were also significantly decreased in HI group indicating worsening locomotion (Fig 8D). These long term findings mimic human PVL on follow up, where they presented with diplegia/paresis of the lower limbs, in-coordination and tendency to fall and with reduced activity and attention.

## Discussion and conclusion

Periventricular leukomalacia (PVL) is a major form of neuropathological brain injury and is the most common cause of cerebral palsy (CP) in premature infants [2]. Currently, there is no available treatment for this devastating injury. Microglia cells have both anti- and pro-inflammatory roles and are recognized as important mediators in the pathogenesis of PVL [1].

Phenotypically, our HI injury mouse model mimics human PVL in several important ways with hind or lower limb paresis, incoordination and initial failure to thrive due to lack of adequate feeding. This model also mimics human PVL histologically in the form of ventriculomegally due to white matter loss from decreased oligodendroglial numbers as well as oligodendroglial maturational arrest. There is also astrogliosis and microgliosis in the periventricular white matter in this model and human PVL.

Cerebral HI injury results in a multifactorial inflammatory cascade that leads to pre- oligodenrocyte (OL) injury, death, and necrosis. The role of specific inflammatory cytokines remains controversial in brain injury of preterm infants,[19] but in HI animal models, IL-1β, IL-6, MIP, IL-9, and TNF-α are believed to play a major role [20]. Anti-inflammatory treatments may represent a useful strategy in ameliorating the effects of PVL, where clinical conditions would favor a post-insult treatment strategy [1]. For example, Lechpammer et al. used minocycline, to suppress microglial activation following HI insult and showed a protective effect against white matter injury and a reduction in microglial cell numbers [1]. Others had shown that attenuation of the pro-inflammatory reaction of astroglia mediated by NF-kB expression using transgenic models with relatively inhibited NF-kB expression, significantly reduced white matter injury in disease models of autoimmune encephalomyelitis and ischemic stroke [21]. In our PVL model, increase phospho p65/p65 activity was significantly higher in HI group as compared to sham indicating an activation of NF-kB pathway along with a significant increase of other inflammatory markers. These inflammatory processes lead to a significant increase of activated microglia (M1 phenotype) and a reduction of repair microglia (M2 phenotype).

Diffuse OL injury in PVL is caused by moderate ischemia. Early differentiating OL is vulnerable to free radical attack, whereas the mature OL is more resistant [2, 22, 23]. OL precursors have deficient antioxidant system. Upon excess free radical exposure to OL precursors, hydrogen peroxide accumulates producing deadly hydroxyl radical which does not happen to mature OL [2,24–26]. In human PVL, the occurrence of free radical injury is supported by evidence of oxidative and nitrative stress with increases in bio-markers of lipid peroxidation and nitrotyrosine [4]. Free radicals are both a cause and a result of inflammation. Reperfusion of ischemic tissues is associated with microvascular injury, due to increased permeability of capillaries and arterioles that lead to an increase of fluid filtration across tissues. These activated injured endothelial cells produce more reactive oxygen and nitrogen species which trigger more inflammatory response. Nitric oxide itself can induce OL damage by two mechanisms: one involving the direct effect of nitric oxide on OL mitochondrial integrity and function, and the other involving an activation of microglia and subsequent release of reactive nitrogen species [26]. Activated microglia/macrophage releases nitric oxide and reactive nitrogen species, which mediate neurotoxicity in several neurodegenerative diseases and hypoxic ischemic insults [27, 28]. In our PVL model, nitrative stress was evident in HI group, mainly inducible and endothelial nitric oxide synthetase (iNOs and eNOS) and nitrotyrosine. The main source of nitrative stress was activated microglia cells as shown in the co-localization of nitrotyrosine to microglia (Fig 7).

It is well established in animal models that ischemia–reperfusion is accompanied rapidly by activation of microglia, secretion of cytokines, and mobilization, adhesion, and migration of macrophages and inflammatory cells [29, 30]. Whether induced by infection or ischemia, these inflammatory responses are detrimental to developing OLs [31–33]. Vasoactive effects of certain cytokines and of nitrogen species released as part of the inflammatory cascade impair cerebrovascular regulation and brain perfusion, thereby increasing the risk for ischemic injury [2,18,34].

In our PVL model, long term neurobehavioral evaluation showed a significant decrease in rear grip strength, overall locomotion and in-coordination mimicking human PVL who present with diplegia and in-coordination. These findings can be explained by periventricular white matter loss as evident in our MRI studies (Fig 2) and histopathological studies (Fig 1) and neuronal apoptosis (Fig 4). MRI studies showed significantly increased ventriculomegally in HI group assessed by ventricle volume and by calculating the ventricle/brain volume.

Collectively, our data demonstrate that in our PVL model NF-kB is activated which could be a cause or a result of activation of pro-inflammatory microglia. Activated microglia triggers a nitrative stress by the release of nitrogen free radicals. Being the most vulnerable to nitrative stress, immature oligodendroglia undergo loss and arrest of development causing white matter loss in our mouse model of PVL.

Our neonate mouse HI model mimics human PVL in symptomatology, pathogenesis, molecular and cellular mechanism and long term neurodevelopmental aspects. The advantages of using this mouse model of PVL is that it mimics human PVL in many ways. Human PVL is usually bilateral and so is this model. Most of the published models of PVL use permanent unilateral carotid artery ligation which produce unilateral PVL. Human PVL usually is not unilateral (although one side may be affected more than the other), and they only suffer temporary and not permanent HI. This model mimics human PVL autopsy specimens in histopathology in terms of white matter loss leading to ventriculomegally, neuronal apotosis, astrogliosis, microglial activation, increased inflammatory markers, and nitrative stress. Our animal model also survives long beyond the HI injury, which allows better long term monitoring and a better understanding of PVL pathogenesis. In addition, a subset of these animals develop cerebral palsy that is often seen in infants affected by PVL. Using mice rather than rats has many advantages including cost effectiveness and the ability to use transgenic animals. The use of a transgenic animal model of PVL will increase our understanding of the mechanistic causes and pathways underlying PVL and permit the development of efficient and targeted treatment and preventative strategies. Thus we believe that we have developed a more appropriate and useful model of PVL that will allow the development of a better understanding of PVL pathogenesis and defining specific targets for the development of suitable therapeutics.
